# Sophflarines F–K: matrine-based alkaloids with a rare aromatic system from *Sophora flavescens* and their anti-inflammatory and synergistic antibacterial effects

**DOI:** 10.1007/s13659-026-00595-2

**Published:** 2026-04-14

**Authors:** Ding Luo, Le-Yan Wang, Xiang-Feng Zhao, Xiao-Lin Huang, Yang-Yi Hu, Qiong-Yun Jing, Yan-Jun Chen, He Tian, Si-Yan Chen, Yu-Yan Wang, Xian-Hui Huang, Zhen-Ling Zeng, Xiao-Ping Liao

**Affiliations:** 1 Guangdong Provincial Key Laboratory of Veterinary Pharmaceutics Development and Safety Evaluation, College of Veterinary Medicine, South China Agricultural University, Guangzhou, 510642 China; 2 National Risk Assessment Laboratory for Antimicrobial Resistance of Animal Original Bacteria, South China Agricultural University, Guangzhou, 510000 China; 3 Department of Anesthesiology, The First Affifiliated Hospital of Jinan University, Guangzhou, 510000 China

**Keywords:** Matrine type alkaloids, *Sophora flavescens*, X-Ray diffraction, Anti inflammatory, Antibacterial synergy

## Abstract

Six new matrine-based alkaloids, sophflarines F–K **(1–6)**, featuring a rare aromatic system, were obtained from the water-soluble alkaloid fractions of *Sophora flavescens* by UV-guided separation. Their structures were elucidated by the interpretation of spectroscopic analyses, quantum chemical calculation, and X-ray diffraction data. Compounds **1** and **2** represent highly modified 15,16-*seco*-17-*nor*-matrine derivatives incorporating a rare 4,5-dihydro-3*H*-pyrrolo[2,3,4-ij]quinolizine moiety, while compound **5** possesses an unusual 6/6/6–5 tetracyclic skeleton. A copper sulfate-induced zebrafish assay revealed that compounds **1**, **4**, and **5** exhibited moderate anti-inflammatory activity at non-toxic concentrations. Checkerboard assays demonstrated that compounds **5** and **6** potentiated colistin activity against *Escherichia coli* ATCC25922 and BW25113-*mcr*-1, reducing colistin MIC values by 16- and 32-fold, respectively. These findings expand the structural diversity of aromatic matrine-type alkaloids and highlight their potential as anti-inflammatory agents and antibacterial adjuvants.

**Graphical Abstract:**

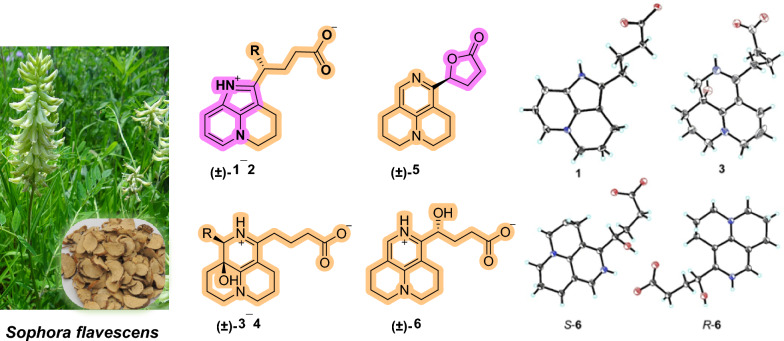

## Introduction

*Sophora flavescens* Ait., a medicinal plant widely distributed throughout East Asia, has been extensively employed in traditional medicine for the treatment of inflammatory conditions, gastrointestinal disorders, and parasitic infections [1–4]. Phytochemical investigations have revealed that this species is abundant in quinolizidine alkaloids, particularly those of the matrine-type [5–7]. These compounds display considerable structural diversity, resulting from various biochemical modifications such as oxidation [8], rearrangement [9, 10], ring-opening [11], and dimerization [12–14]. Such structural variations underpin a broad range of pharmacological properties, including anti-inflammatory [15, 16], antiviral [17], antibacterial [3, 18, 19], anti-fibrotic [20], and immunomodulatory activities [21]. Despite this diversity, most matrine-type alkaloids are fully saturated and sp^3^-rich, showing only terminal UV absorption; by contrast, aromatic and highly modified matrine derivatives are rare, with only a few structural types reported, yet they have attracted increasing interest due to their distinctive scaffolds and promising bioactivities [7, 11].

**Fig. 1:**
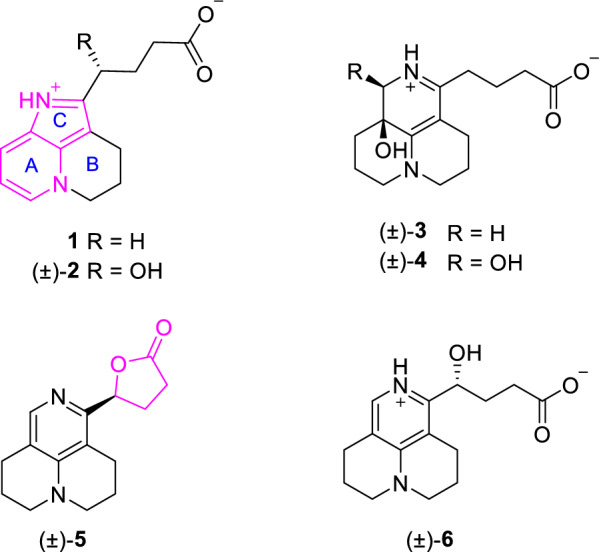
Chemical structures of compounds **1−6**

Inflammation and bacterial drug resistance remain serious global health concerns [22, 23]. Excessive inflammatory responses contribute to the progression of many infectious and chronic diseases, while antimicrobial resistance limits the effectiveness of existing antibiotics [24, 25]. As a result, combination therapy has become an important approach to enhance antibacterial efficacy. As part of our ongoing investigation of bioactive alkaloids from *S. flavescens *[7, 26], we isolated six rare aromatic matrine-based alkaloids, designated sophflarines F–K (Fig. 1). Compounds **1** and **2** represent the first *nor*-matrine derivatives featuring a 4,5-dihydro-3*H*-pyrrolo[2,3,4-ij]quinolizine moiety and an aromatic A/C ring, which is clearly distinct from previously reported *seco*- or *nor*-matrine scaffolds that mainly involve simple ring cleavage without extensive skeletal reorganization. This paper details the isolation, structural elucidation, plausible biosynthetic pathway, anti-inflammatory and synergistic antibacterial activities of these alkaloids.

## Results and discussion

### Isolation and structural identification

The total alkaloids were prepared from *S. flavescens* roots via a standard acid–base extraction. During preliminary profiling of the alkaloid extract, several constituents displayed a UV absorption band near 350 nm, indicative of an extended conjugated system rarely encountered in matrine-type alkaloids [27]. This diagnostic spectral feature enabled the UV-guided isolation of compounds **1–6**.

**Fig. 2:**
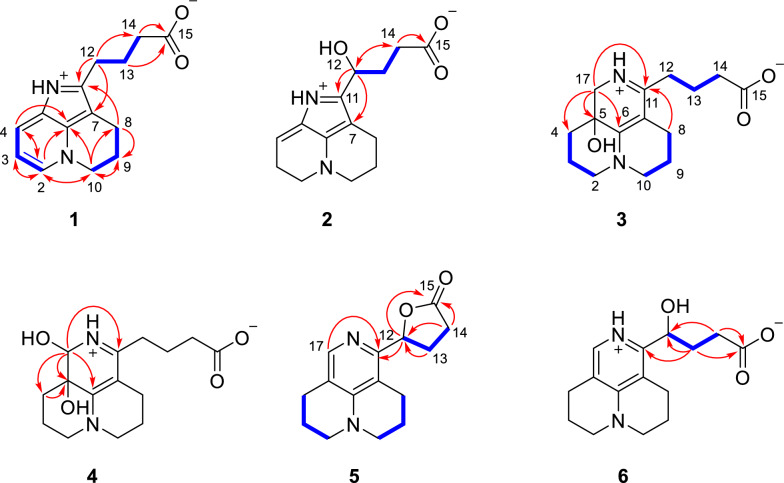
Key ^1^H-^1^H COSY, HMBC, and NOESY correlations of compounds **1−6**

**Fig. 3:**
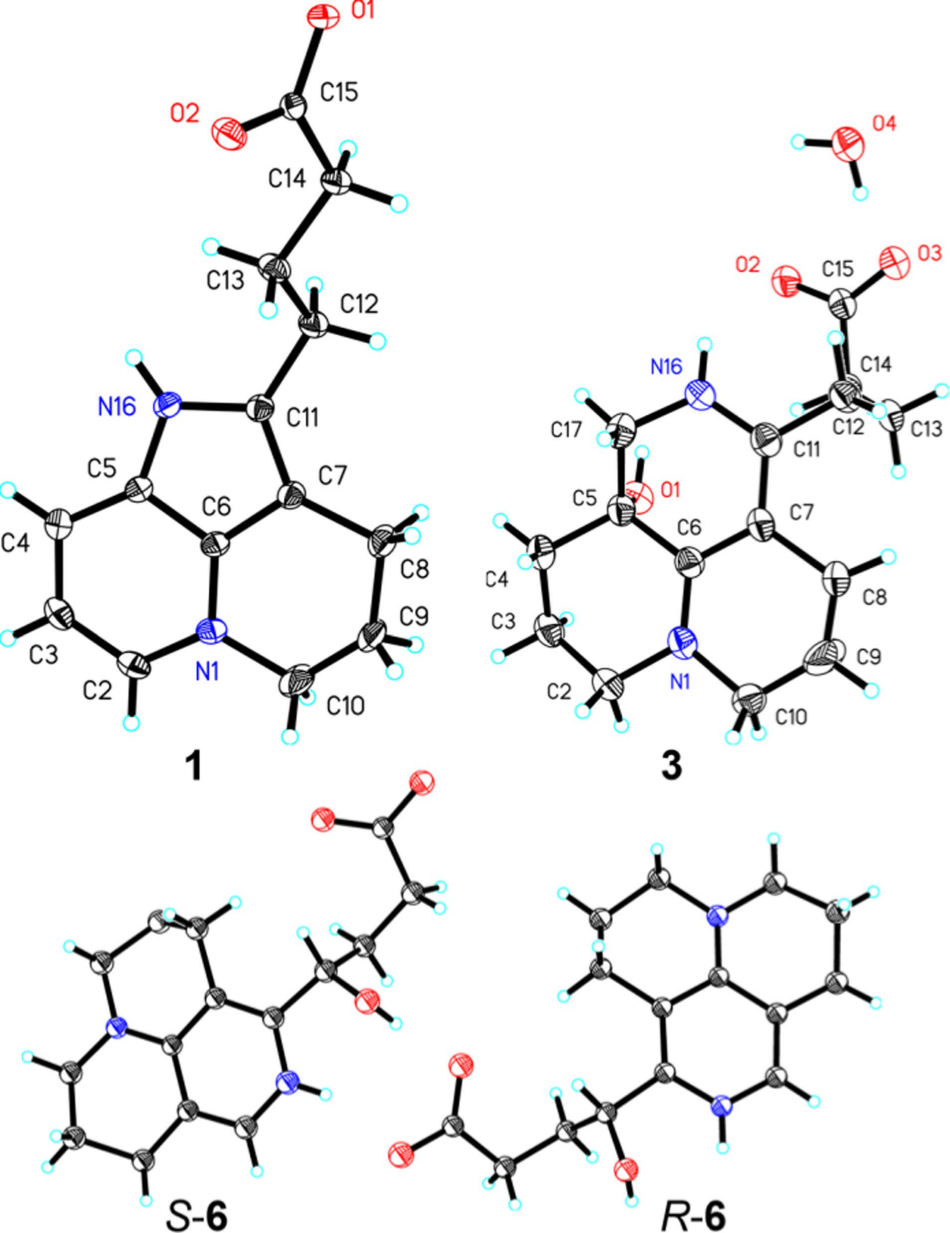
X-ray crystal structures of compounds **1**, **3**, and (±)-**6** (ORTEP drawings at 30% probability ellipsoids)

Compound **1** was isolated as pale yellow crystals with negligible optical rotation value. The UV spectra exhibited an unusual conjugated chromophore absorption maxima at *λ*_max_ 223, 237, 291, and 352 nm. The HRESIMS ion peak gave a molecular formula of C_14_H_16_N_2_O_2_ ([M + H]^+^ at 245.1282, calcd for C_14_H_17_N_2_O_2_, 245.1285), requiring 8 indices of hydrogen deficiency (IHDs). The ^1^H NMR spectrum showed the signals for 1,2-disubstituted pyridine ring [*δ*_H_ 8.38 dd (6.5, 2.6), 8.24 dd (8.0, 2.6), 7.49 dd (8.0, 6.5)] and several *sp*^3^ methylene emerging at *δ*_H_ [4.68 m, 3.00 m, 3.97 m, 2.38 m, 2.24 td (7.3, 2.5), and 2.08 m], respectively. The ^13^C NMR and DEPT data showed a C_14_ backbone assignable to a carboxy carbon (*δ*_C_ 180.0), seven aromatic carbons (*δ*_C_ 148.3, 137.0, 133.6, 131.2, 124.1, 115.6, 104.4), and six methylene (*δ*_C_ 51.6, 36.8, 26.3, 25.4, 22.1, 17.5) (Table 1). These mentioned spectroscopic data together with biosynthetic reasoning revealed a *nor*-matrine incorporating terminal carboxyl side chain and unusual aromatic system, similar to flavesine G, which also displayed a ring D-*seco* and aromatic C ring [11]. The body ring structural unit that fused via C-5 − C-6 − C-7 was established by ^1^H-^1^H COSY spin system of H-2/H-3/H-4 and H_2_-8/H_2_-9/H_2_-10, and multiple HMBC correlations from H-2 to C-3/C-4/C-6/C-10, from H_2_-8 to C-9/C-10/C-11, and from H_2_-10 to C-2/C-6/C-8/C-9 (Fig. 2). The side chain attached at C-11 was deduced from key HMBC correlations from H_2_-12 to C-7/C-11. The 2D structure of **1** was verified by X-ray crystallographic data with unique C_2_/c space group (Fig. 3). Ultimately, the chemical structure of **1** was identified to be an unprecedented matrine-based skeleton involving 15,16-*seco*, ring-aromatization and 17-*nor* structural modification, named sophflarine F.

**Table 1:** ^1^H and ^13^C NMR data of **1–2** (CD_3_OD, *δ* in ppm, *J* in Hz)^*a,b*^

No	**1**	*δ*_H_	**2**	*δ*_H_
	*δ*_C_, Type		*δ*_C_, Type	
2	133.6, CH	8.38 (6.5, 2.6)	135.4, CH	8.38 d (6.0)
3	115.6, CH	7.49 (8.0, 6.5)	117.3, CH	7.51 dd (8.0, 6.0)
4	124.1, CH	8.24 (8.0, 2.6)	126.2, CH	8.26 d (8.0)
5	131.2, C		132.3, C	
6	137.0, C		138.1, C	
7	104.4, C		104.7, C	
8	17.5, CH_2_	2.97 m	19.4, CH_2_	3.04 m
9	22.1, CH_2_	2.38 m	23.5, CH_2_	2.36 m
10	51.6, CH_2_	4.68 m	53.1, CH_2_	4.69 m
11	148.2, C		151.2, C	
12	25.4, CH_2_	2.08 m	68.5, CH	5.13 dd (6.8, 5.6)
13	26.2, CH_2_	3.00 m	34.6, CH_2_	2.17 m
14	36.8, CH_2_	2.24 td (7.3, 2.5)	34.8, CH_2_	2.30 m
15	180.0, C		181.6, C	

^*a*^Overlapped signals were reported without designating multiplicity

^*b*^Recorded at 600 MHz for ^1^H and 150 MHz for ^13^C

The only difference between **2** and **1** was the substitution of C-12 methylene [(*δ*_C_ 25.4, *δ*_H_ 2.08 (2H, overlaped)] in **1** by a hydroxylated methine (*δ*_C_ 71.6, *δ*_H_ 4.95) in **2**, as supported by the HMBC correlations from H-12 → C-7/C-11/C-14 and H_2_-14 → C-12/C-15 (Fig. 2). Its smooth ECD curve and specific rotation near 0 indicated the racemic nature (Fig. S12). The chiral resolution of **2** was unsuccessful because of the rapid rotation of its flexible side chain. Since **2** has only one chiral center at C-12, the 12*R* or 12*S* configuration correspond to positive or negative value of the optical rotation, respectively. Therefore, the relationship between the positive/negative theoretical optical rotation values and the 12*R*/12*S* absolute configuration was established by optical rotation calculation on three different basis sets (Table 2). The enantiomers of **2** was finally designated as (+)-12*R*-sophflarine G and (−)-12*S*-sophflarine G, respectively.

**Table 2:** Calculated optical rotation values of compounds **2**, **5**, and **6** (in deg [dmg/cm^3^]^−1^)

DFT method/Basis set	***R* − 2**	***S* − 5**	***R* − 6**
B3LYP/6-31G(d)	+ 43.5	+ 300.9	+ 156.8
B3LYP/cc-pVDZ	+ 40.1	+ 306.0	+ 152.6
B3LYP/6–311 + + G(2d,p)	+ 122.8	+ 288.0	+ 123.5

**Fig. 4:**
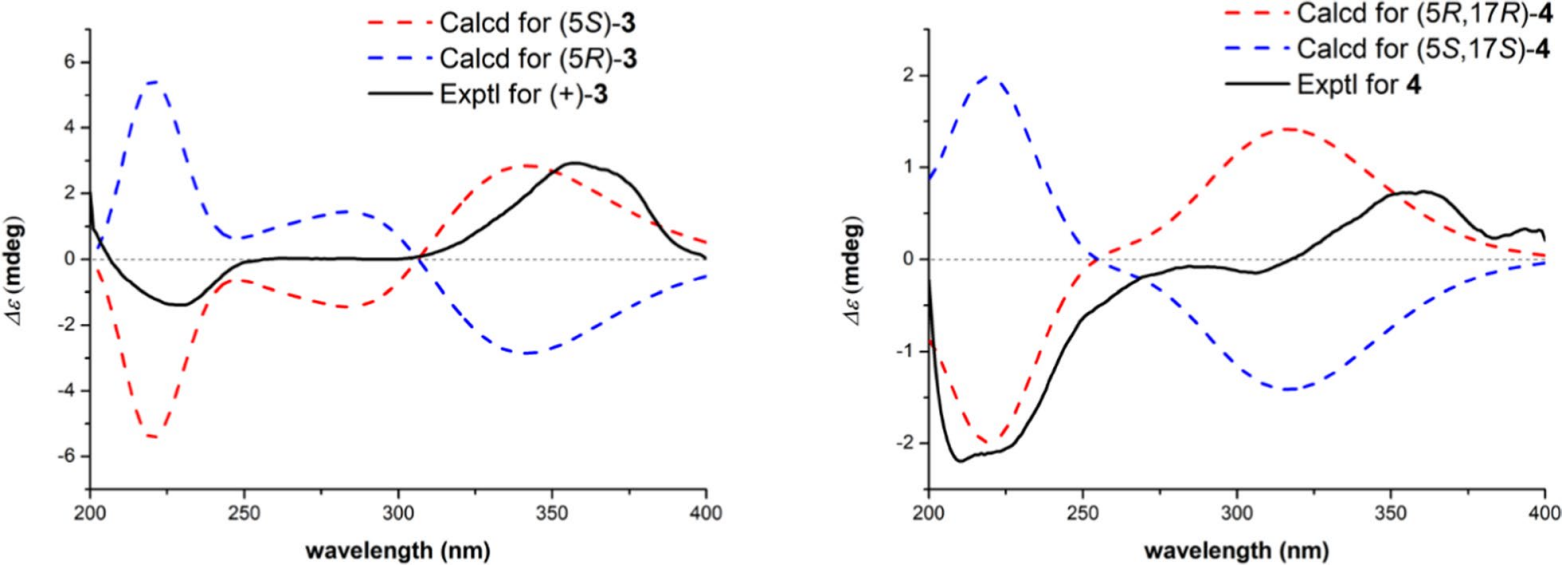
Calculated or experimental ECD spectra of compounds **3–4**

Compound **3** was obtained as colorless crystals and had a molecular formula of C_15_H_22_N_2_O_3_ [*m/z:* 279.1694 [M + H]^+^ (calcd for C_15_H_23_N_2_O_3_, 279.1703)], requiring six IHDs. The NMR data of **3** exhibited similar chemical shifts to those of flavesine I [28], indicating the presence of a rare 15, 16-*seco*-matrine skeleton (Table 3). The key difference was that **3** has an additional hydroxy group at C-5 than flavesine I, based on its missing COSY correlations of H_2_-4/H-5 and H_2_-17/H-5, together with HMBC correlations from H_2_-17 to C-4/C-5/C-6/C-11 and from H_2_-4 to C-2/C-5/C-17 (Fig. 2). Furthermore, the gross structure of **3** was definitively identified by X-ray crystallographic data (Fig. 3). The crystal of **3** has the space group P2_1_/n and near 0 optical rotation indicating it existed in racemic form. Subsequent chiral HPLC separation of **3** was performed to afford a pair of enantiomers with 1:1 ratio (Fig. S22). The positive optical rotation value of (+)**-3**, [*α*]_D_^25^ 130.7 (*c* 0.01, CH_3_OH), with experimental ECD curve dominated positive Cotton effects 355 nm and negative ellipticity around 220 nm consistent with 5*S*-**3** conformer (Fig. 4). Thus, the absolute configurations of both enantiomers were determined as (+)-5*S*-sophflarine H and (−)-5*R*-sophflarine H.

**Table 3:** ^1^H and ^13^C NMR data of **3–6** (CD_3_OD, *δ* in ppm, *J* in Hz)^*a,b*^

No	**3**		**4**		**5**		**6**	
	*δ*_C_, type	*δ*_H_	*δ*_C_, type	*δ*_H_	*δ*_C_, type	*δ*_H_	*δ*_C_, type	*δ*_H_
2	52.4, CH_2_	3.45 m	53.2, CH_2_	3.47 m	51.0, CH_2_	3.49 m	51.3, CH_2_	3.45 m
3	18.3, CH_2_	a 2.36; b 1.87	18.5, CH_2_	a 2.35; b 1.88	20.9, CH_2_	1.99	20.9, CH_2_	1.97
4	30.4, CH_2_	a 1.63 m; b 1.85	28.3, CH_2_	a 1.72 dt (14.0, 3.5); b 2.10 td (14.0, 3.5)	25.3, CH_2_	2.77 m	25.1, CH_2_	2.74
5	64.3, C		68.0, C		117.8, C		117.2, C	
6	161.4, C		162.7, C		152.8, C		153.2, C	
7	95.6, C		95.1, C		112.8, C		113.4, C	
8	22.1, CH_2_	a 2.52; b 2.40	21.8, CH_2_	2.43	23.7, CH_2_	2.69 m	22.7, CH_2_	2.73
9	21.2, CH_2_	1.93	21.2, CH_2_	1.95	20.5, CH_2_	1.99	20.6, CH_2_	1.97
10	52.1, CH_2_	3.45 m	52.7, CH_2_	3.47 m	50.4, CH_2_	3.49 m	50.5, CH_2_	3.45 m
11	169.2, C		167.0, C		152.1, C		151.9, C	
12	33.5, CH_2_	a 2.53 m; b 2.45 m	32.8, CH_2_	2.52 m	71.6, CH	4.95 m	68.9, CH	4.96 dd (8.3, 3.4)
13	25.1, CH_2_	1.88	25.0, CH_2_	1.87 m	28.3, CH_2_	a 2.63; b 2.45	33.6, CH_2_	a 2.00 m; b 1.85 m
14	37.9, CH_2_	2.24 t (7.0)	37.9, CH_2_	2.25 t (7.1)	29.7, CH_2_	3.11	34.9, CH_2_	2.33 m
15	181.3, C		181.5, C		175.4, C		181.7, C	
17	51.9, CH_2_	3.48 m	80.4, CH	4.44 s	136.1, CH	7.77 s	135.9, CH	7.63 s

^*a*^Overlapped signals were reported without designating multiplicity

^*b*^Recorded at 600 MHz for ^1^H and 150 MHz for ^13^C

HRESI(+)MS measurements on **4** revealed a molecular formula C_15_H_22_N_2_O_4_ suggestive of an oxidized (+ O) homologue of **3**. Comparison of the NMR data for **4** with **3** revealed the principal differences as transformation of the C-17 methylene in 3 into a hydroxy substituent methine in **4** (Table 3). Confirming evidence was obtained from the downfield chemical shifts of C-17 (*δ*_C_ 51.9 → 80.4) and the HMBC correlations of protons from H-17 (*δ*_H_ 4.44) to C-4/C-5/C-6/C-11 (Fig. 2). Inspired by the conformation provided by the X-ray crystal structure of **3**, the ring A of **4** was thought to adopt a chair conformation and H-4a was subsequently identified as being on the axial bond (*δ*_H__eq_ > *δ*_H__aq_). Therefore, the observed NOE between H-4a and H-17 indicates that OH-5 and OH-17 are on the same face of the molecule, as shown in computer generated 3D drawing (Fig. S44). The CD spectrum of **4** was weak (non-racemic mixture in 1:1), but its overall shape matched best with the calculated spectrum of the (5*R*,17*R*)-configuration, indicating that the sample of 4 was enriched in (5*R*,17*R*)-**4** (Fig. 4). Finally, the absolute configurations of **4** were tentatively assigned as (+)-(5*R*,17*R*)-sophflarine I and (−)-(5*S*,17*S*)-sophflarine I.

HRESIMS established the molecular formulas of **5** and **6** as C_15_H_18_N_2_O_3_ and C_15_H_20_N_2_O_3_, respectively. For compound **6**, comparison with flavesine G showed that the sole structural modification was the replacement of the C-12 methylene by a hydroxylated methane [11], supported by the HMBC cross-peaks H-12 → C-7/C-11/C-14 and H₂-14 → C-12/C-15 (Fig. 2). Single-crystal X-ray diffraction further confirmed the planar structure of **6** and revealed a P1 space group containing both (*R*)- and (*S*)-6 within the asymmetric unit (Fig. 3). Compound **5** displayed NMR data highly similar to those of **6**, yet detailed MS/NMR comparison revealed that **5** possesses one additional degree of unsaturation. The characteristic shifts at C-12 (*δ*_C_ 68.9 → 71.6) and C-14 (*δ*_C_ 181.7 → 175.4) are diagnostic for intramolecular lactonization [29], as ester formation typically shields the carbonyl carbon and deshields the oxygenated methine. This transformation was corroborated by the COSY sequence H-12 ↔ H₂-13 ↔ H₂-14, indicating a transition from a flexible side chain to a constrained ring system, and by the key HMBC correlation H-12 → C-11/C-13/C-15, confirming a five-membered γ-lactone in **5**. Finally, because both compounds displayed poor resolution on chiral HPLC columns, chiral separation was not attempted. Analogous to compound **2**, optical rotation values were calculated using single-point B3LYP calculations with different basis sets (6-31 G(d), cc-pVDZ, and 6–311 +  + G(2d,p)). Based on these results, the configurations of the enantiomers were assigned as (+)-(12*S*)-**5** and (−)-(12*R*)-**5**, and (+)-(12*R*)-**6 **and (−)-(12*S*)-**6**, respectively (Table 2).

**Scheme 1:**
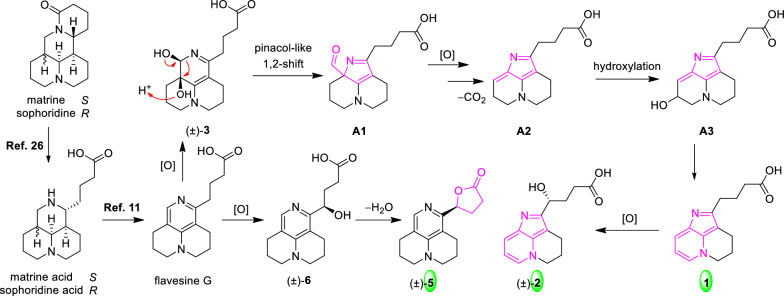
Plausible biosynthetic proposal for compounds **1−2**, and **5**

Plausible biogenetic pathways for compounds **1–2** and **5** were proposed based on the co-isolated analogues, as illustrated in Scheme 1. All of these metabolites can be hypothetically traced back to sophoridine or matrine, two major quinolizidine alkaloids that together account for more than 50% of the crude alkaloid fraction [30]. On the upper pathway, the co-isolated compound **3** may first undergo an acid-promoted pinacol-like 1,2-shift, affording the rearranged intermediate **A1** [31]. Subsequent oxidation followed by decarboxylation would furnish intermediate **A2**, which can be further converted into **A3** via hydroxylation [32]. A dehydration step from **A3** then establishes the characteristic aromatic C-ring system observed in **1**. Additional hydroxylation at C-12 of **1** gives rise to compound **2**. On the lower pathway, oxidation of flavesine G generates the corresponding carboxyl intermediate, which upon dehydration affords the γ-lactone scaffold [33], thereby yielding compound **5**.

### In vivo anti-inflammatory assay

**Fig. 5:**
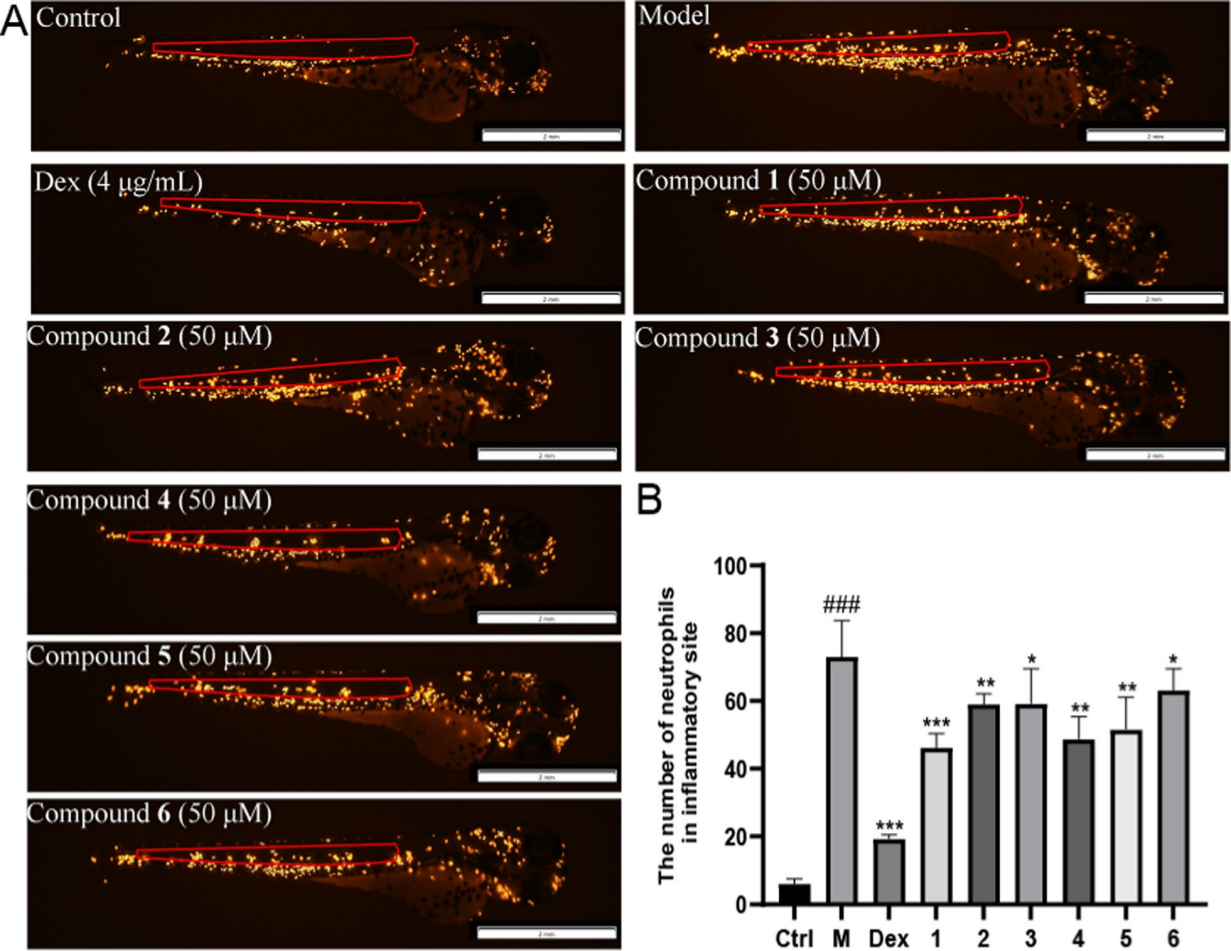
Anti-inflammatory effects of compounds **1−6** in zebrafish inflammatory models. **A** Representative images of zebrafish treated with different isolates in zebrafish inflammatory models induced by CuSO_4_. **B** Neutrophils in the red region were quantitatively analyzed. Data were represented as mean ± SEM from three independent experiments. Statistical analysis was performed using Student’s t-test. *P < 0.05, **P < 0.01, ***P < 0.001 vs. the model group. ^###^*P* < 0.001 *vs*. the control group. *Ctrl* control group, *M* Model, *Dex* dexamethasone group

Inspired by the results from previous assays [34, 35], the in vivo anti-inflammatory activity of compounds **1–6** was evaluated in the Copper sulfate-induced zebrafish inflammation model (Fig. 5). Compounds **1, 4, and 5** displayed moderate anti-inflammatory effects at their non-toxic concentration (50 μM), as reflected by the decreased fluorescence intensity around the neuromasts.

### Synergistic antibacterial activity with colistin

Plasmid-mediated colistin resistance, particularly driven by *mcr* genes, has become a major challenge in the treatment of multidrug-resistant Gram-negative infections, and combination therapy is considered an effective strategy for restoring colistin susceptibility [23, 36, 37]. The synergistic effects of colistin in combination with compounds **1–6** were evaluated using checkerboard assays against *E. coli* ATCC25922 and BW25113-*mcr*-1 (Table 4). In *E. coli* ATCC25922, compounds **1**, **2**, **5**, and **6** markedly enhanced colistin susceptibility, lowering its MIC from 4 μg·mL^−1^ to 1 and 0.125 μg·mL^−1^ (a 4- to 32-fold reduction). Importantly, compounds **2**, **5**, and **6** also restored colistin activity against the *mcr*-1–positive strain BW25113, reducing its MIC by 4–32 fold and yielding synergistic FICI values.

**Table 4:** MIC and FIC values of colistin alone or in combination with compounds **1–6** against *E. coli*^*a*^

Bacterial strains	MIC of monotherapy (μg/mL)			MIC of combination (μg/mL)	FICI	Outcome (Fold-changes of antibiotic)
	Antibiotic	Agent		Antibiotic/Agent		
ATCC 25922	Colistin, 4 μg/mL	**1**	> 512	1/128	0.25	Synergy (4 ×)
		**2**	> 512	0.5/128	0.156	Synergy (8 ×)
		**3**	> 512	2/256	0.75	Additive (2 ×)
		**4**	> 512	4/512	2.0	Indifferent (1 ×)
		**5**	> 512	0.125/128	0.127	Synergy (32 ×)
		**6**	> 512	0.25/64	0.133	Synergy (16 ×)
BW25113-mcr-1	Colistin, 4 μg/mL	**1**	> 512	2/256	0.75	Additive (2 ×)
		**2**	> 512	1/256	0.50	Synergy (4 ×)
		**3**	> 512	2/512	1.0	Additive (2 ×)
		**4**	> 512	4/512	2.0	Indifferent (1 ×)
		**5**	> 512	0.125/256	0.264	Synergy (32 ×)
		**6**	> 512	0.25/64	0.133	Synergy (16 ×)

^*a*^For compounds with MICs reported as “ > 512 μg/mL”, a value of 512 μg/mL was used for FIC calculations

## Experimental

### General experimental procedures

The melting points of crystalline samples were determined on an X-5 digital micro-melting point apparatus and are presented without correction. UV spectra were obtained using a JASCO V-550 UV/VIS spectrophotometer, and optical rotations were measured on an Autopol JASCO P-1020 digital polarimeter. IR spectra were recorded on a JASCO FT/IR-480 Plus spectrometer using KBr pellets. NMR measurements, including 1D and 2D experiments, were performed on a Bruker Avance 600 spectrometer (600 MHz for ^1^H and 150 MHz for ^13^C) equipped with standard Bruker pulse programs. Chemical shifts (*δ*) were expressed in ppm with reference to residual solvent peaks. High-resolution electrospray ionization mass spectra (HR-ESI–MS) were acquired using a Waters Synapt G2 mass spectrometer. Single-crystal X-ray diffraction data were collected on an Agilent Gemini Ultra diffractometer fitted with a Cu-Kα radiation source (λ = 1.54178 Å) and a CCD area detector (Agilent Technologies, USA). HPLC analyses were conducted on a Shimadzu system (Shimadzu Corporation, Tokyo, Japan) equipped with a PDA detector, using analytical or preparative Waters XBridge RP18 columns (MeCN/H₂O or MeOH/H₂O as eluents). TLC analysis employed aluminium oxide 60 F₂₅₄ basic plates (Merck, China). The chromatographic materials used in this study included D-101 macroporous resin (Diaion, Shanghai, China), neutral alumina N (200–300 mesh; Aldrich, China), Sephadex LH-20 (25–100 μm; Fluka, Switzerland), MCI CHP20P gel (75–150 μm; Sigma-Aldrich, China), and ODS silica gel (50 μm; YMC, Japan). All solvents and reagents were of analytical grade and purchased from commercial suppliers in China.

### Plant material

The roots of *S. flavescens* Ait. were collected in September 2019 from Xi’an, Shaanxi Province, China (34°46′–34°55′ N, 109°08′–109°16′ E). The botanical identity of the material was verified by Mr. Zhengqiu Mai, a senior herbal specialist at the Chinese Medicinal Material Co. (Guangzhou, China). A voucher specimen (No. SF-201909) is deposited at the Guangdong Provincial Key Laboratory of Veterinary Pharmaceutics Development and Safety Evaluation, College of Veterinary Medicine, South China Agricultural University, Guangzhou, China.

### Extraction and isolation

The powdered roots of *S. flavescens* (25 kg) were extracted three times with 95% EtOH (48 h each), and the combined extracts were concentrated to yield a crude ethanol extract (~ 3.5 kg). Suspension of the crude extract in water followed by acidification to pH 3–4 with dilute HCl enabled the removal of neutral constituents through extraction with CHCl₃. Basification of the remaining aqueous phase to pH 9–10 with saturated NH₄OH, followed by extraction with CHCl₃, afforded the crude alkaloid extract (926 g, extraction coefficient: 3.7%). The alkaloid extract was chromatographed on a D-101 macroporous resin column eluted with graded EtOH–H₂O mixtures (10%–95%), yielding five fractions designated Fr.1–Fr.5. Fraction Fr.1 was dissolved in water and partitioned twice with EtOAc to remove lipophilic materials, and the aqueous-enriched portion was collected as Fr.1A (60.0 g). Chromatography of Fr.1A on neutral Al₂O₃ using CH₂Cl₂–MeOH (100:0 → 0:100, *v/v*, with 1% Et₂NH) yielded seven subfractions, Fr.1Aa–Fr.1Ag. Among these, Fr.1Ag was further purified on an MCI CHP20P column eluted with MeOH–H₂O–NH₄OH to obtain sixteen fractions (Fr.1Ag.1–Fr.1Ag.16). Fractions Fr.1Ag.1–Fr.1Ag.5 were successively subjected to Sephadex LH-20 chromatography (MeOH–H₂O, 1:1) to enrich minor alkaloids. The combined eluates were chromatographed on an ODS column with increasing proportions of MeOH–H₂O (5%–50%) to obtain Fr.1Ag.1–5. Final purification was carried out by semipreparative HPLC on a Waters XBridge^™^ Phenyl column with MeOH/H₂O or CH₃CN/H₂O (each containing 0.1% Et₂NH; isocratic or shallow gradients as appropriate), affording compounds **1** (88.8 mg, t_R_ = 9.7 min), **2** (0.9 mg, t_R_ = 6.4 min), **3** (54.2 mg, t_R_ = 16.1 min), and **4** (33.9 mg, t_R_ = 21.5 min). Compounds **5** (7.0 mg, t_R_ = 31.0 min) and **6** (69.9 mg, t_R_ = 19.0 min) were purified by preparative RP-HPLC using CH₃CN/H₂O/Et₂NH (15:85:0.01, *v/v/v*).

### Spectroscopic data of the isolates

*Sophflarine F* **(1)**: pale yellow crystals in CH_3_OH; m.p. 128–129 °C; [*α*]_D_^25^ ± 0 (*c* 0.01, CH_3_OH); UV (CH_3_OH) *λ*_max_ (log *ε*) 223 (3.17), 237 (3.02), 291 (2.66), and 352 (2.61) nm; IR (KBr) *ν*_max_ 3412, 3080, 2937, 2859, 1561, 1447, 1389, 1041 cm^−1^; ^1^H NMR (600 MHz, CD_3_OD) and ^13^C NMR (150 MHz, CD_3_OD), see Table 1; HRESIMS *m/z* 245.1282 [M + H]^+^ (calcd for C_14_H_17_N_2_O_2_, 245.1285).

*Sophflarine G* **(2)**: pale yellow oil; [*α*]_D_^25^ ± 0 (*c* 0.01, CH_3_OH); UV (CH_3_OH) *λ*_max_ (log *ε*) 200 (3.17), 223 (2.92), 290 (2.71) and 351 (2.61) nm; IR (KBr) *ν*_max_ 3390, 2931, 2859, 1593,1511, 1415, 1036 cm^−1^; ^1^H NMR (600 MHz, CD_3_OD) and ^13^C NMR (150 MHz, CD_3_OD), see Table 1; HR-ESI–MS *m/z*: 261.1235 [M + H]^+^ (calcd for C_14_H_17_N_2_O_3_, 261.1234).

*Sophflarine H* **(3)**: colorless crystals in CH_3_OH; mp 135–136 °C; [*α*]_D_^25^ ± 0 (*c* 0.01, CH_3_OH); UV (CH_3_OH) *λ*_max_ (log ε) 201 (3.33) and 359 (2.91) nm; IR (KBr) *ν*_max_ 3423, 2929, 2857, 1632, 1451, 1415, 1331, 1162 cm^−1^ ^1^H NMR (600 MHz, CD_3_OD) and ^13^C NMR (150 MHz, CD_3_OD), see Table 3; HR-ESI–MS *m/z*: 279.1694 [M + H]^+^ (calcd for C_15_H_23_N_2_O_3_, 279.1703); *(* +*)-Sophflarine H:* white amorphous powder; [*α*]_D_^25^ 130.7 (*c* 0.01, CH_3_OH); ECD (CH_3_OH) *λ*_max_ (Δ*ε*) 233 (− 1.4), 357 (+ 2.9) nm; *( −)-Sophflarine H:* white amorphous powder; [*α*]_D_^25^ − 135.5 (*c* 0.01, CH_3_OH); ECD (CH_3_OH) *λ*_max_ (Δ*ε*) 233 (− 13.2), 357 (+ 28.5) nm.

*Sophflarine I* **(4)**: brown oil; [*α*]_D_^25^ 3.3 (*c* 0.01, CH_3_OH); UV (CH_3_OH) *λ*_max_ (log ε) 200 (3.18) and 359 (3.01) nm; ECD (CH_3_OH) *λ*_max_ (Δ*ε*) 233 (− 1.4), 357 (+ 2.9) nm. IR (KBr) *ν*_max_ 3443, 3287, 2922, 2854, 1629, 1470, 1411, 1330 cm^−1^; ^1^H NMR (600 MHz, CD_3_OD) and ^13^C NMR (150 MHz, CD_3_OD), see Table 3; HR-ESI–MS *m/z*: 295.1642 [M + H]^+^ (calcd for C_15_H_23_N_2_O_4_, 295.1652).

*Sophflarine J* **(5)**: pale yellow oil; [*α*]_D_^25^ 6.3 (*c* 0.01, CH_3_OH); UV (CH_3_OH) *λ*_max_ 200 (3.13), 308 (2.68) nm; ECD (CH_3_OH) *λ*_max_ (Δ*ε*) 213 (+ 0.3), 297 (− 3.7), 340 (+ 1.7) nm. IR (KBr) *ν*_max_ 2933, 2866, 1625, 1603, 1444, 1332 cm^−1^; ^1^H NMR (600 MHz, CD_3_OD) and ^13^C NMR (150 MHz, CD_3_OD), see Table 3; HR-ESI–MS *m/z*: 259.1439 [M + H]^+^ (calcd for C_15_H_19_N_2_O_2_, 259.1441).

*Sophflarine K* **(6)**: brown oil; [*α*]_D_^25^ ± 0 (*c* 0.01, CH_3_OH); UV (CH_3_OH) *λ*_max_ 205, 228, and 296 nm; IR (KBr) *ν*_max_ 3443, 3287, 2922, 2854, 1629, 1470, 1451 cm^−1^; ^1^H NMR (600 MHz, CD_3_OD) and ^13^C NMR (150 MHz, CD_3_OD), see Table 3; HR-ESI–MS *m/z*: 277.1520 [M + H]^+^ (calcd for C_15_H_21_N_2_O_3_, 277.1547).

### *X-ray crystallographic analyses of compounds *1, 3, and 6

Single-crystal X-ray diffraction analyses of compounds **1**, **3**, and **6** were performed using an Agilent Gemini Ultra diffractometer equipped with a CCD area detector and employing Cu Kα radiation (λ = 1.54184 Å). Structural determination was conducted using direct methods via the SHELXT program, followed by full-matrix least-squares refinement on F^2^ using SHELXL or SHELXS within the Olex2 platform. The crystallographic information files (CIFs) for compounds **1**, **3**, and **6** have been deposited in the Cambridge Crystallographic Data Centre (CCDC, https://www.ccdc.cam.ac.uk/) under deposition numbers 2085308, 2,358,218, and 2,358,219, respectively. ORTEP representations of the crystal structures were generated using SHELXP. A summary of crystallographic data and refinement parameters is provided below:

Crystal data for sophflarine F **(1)**: C_14_H_20_N_2_O_4_ (*M* = 280.32 g/mol), monoclinic, space group C2/c (no. 15), *a* = 50.0093(10) Å, *b* = 11.3394(2) Å, *c* = 14.5656(3) Å, *β* = 90.749(2)°, *V* = 8259.1(3) Å^3^, *Z* = 24, *T* = 149.99(10) K, μ(CuKα) = 0.823 mm^−1^, *Dcalc* = 1.353 g/cm^3^, 16,655 reflections measured (7.072° ≤ 2Θ ≤ 147.84°), 8107 unique (*R*_int_ = 0.0247, R_sigma_ = 0.0310) which were used in all calculations. The final *R*_1_ was 0.0754 (I > 2σ(I)) and *wR*_2_ was 0.1958 (all data). The goodness of fit on *F*^2^ was 1.080. CCDC number: 2085308.

Crystal data for sophflarine H **(3)**: C_15_H_22_N_2_O_4_ (*M* = 258.30 g/mol): monoclinic, space group P2_1_/n (no. 14), *a* = 8.340(2) Å, *b* = 14.179(4) Å, *c* = 12.361(3) Å, *β* = 98.75(3)°, *V* = 1444.8(7) Å^3^, *Z* = 4, *T* = 293(2) K, *μ*(CuKα) = 0.611 mm^−1^, *D*_*calc*_ = 1.187 g/cm3, 5060 reflections measured (9.556° ≤ 2*Θ* ≤ 151.168°), 2839 unique (*R*_int_ = 0.0519, R_sigma_ = 0.0601) which were used in all calculations. The final *R*_1_ was 0.1255 (*I* > *2σ(I)*) and *wR*_2_ was 0.3621 (all data). The goodness of fit on *F*^2^ was 0.865. CCDC number: 2358218.

Crystal data for sophflarine K **(6)**: C_15_H_23_N_2_O_4.5_ (*M* = 303.35 g/mol): triclinic, space group P-1 (no. 2), *a* = 8.7462(8) Å, *b* = 11.2707(8) Å, *c* = 16.4281(11) Å, *α* = 81.481(6)°, *β* = 78.275(7)°, *γ* = 68.629(8)°, *V* = 1471.7(2) Å^3^, *Z* = 4, *T* = 293(2) K, μ(CuKα) = 0.836 mm^−1^, *Dcalc* = 1.369 g/cm^3^, 10,848 reflections measured (5.512° ≤ 2Θ ≤ 147.798°), 5777 unique (*R*_int_ = 0.0433, R_sigma_ = 0.0562) which were used in all calculations. The final *R*_1_ was 0.0958 (I > 2σ(I)) and *wR*_2_ was 0.2977 (all data). The goodness of fit on *F*^2^ was 1.076. CCDC number: 2358219.

### Quantum chemical ECD and ORD calculations

Theoretical ECD calculations of compounds **3–4** and optical rotation calculations of compounds **2** **5**, and **6** were performed using the Gaussian 09 software package. Conformational analysis was initially conducted using the Sybyl-X 2.1.1 program with an energy cutoff of 10 kcal/mol relative to the global minimum. These conformers were further optimized at the DFT/B3LYP/6-31G(d,p) level. For ORD calculations, single-point optical rotation calculations were performed at the B3LYP level using different basis sets, including 6-31G(d), cc-pVDZ, and 6–311 +  + G(2d,p), based on the optimized conformers. The final optical rotation values reported in Table 3 represent Boltzmann-weighted averages derived from the corresponding conformer populations. ECD spectra were simulated from the first 50 singlet electronic transitions using Gaussian band shapes with a standard deviation of 0.33 eV [38]. Additional experimental details can be found in our previous report [6]. Optimized Cartesian coordinates, thermodynamic data, and conformer populations for compounds **2–6** are provided in the Supporting Information.

### *CuSO*_*4*_*-induced inflammation and drug treatments*

CuSO_4_-induced inflammation assay was performed based on our previous report. Briefly, 3 days post fertilization (dpf), healthy larvae were immersed in 20 μM CuSO_4_ solution containing sophflarines F− K (50 μM). The behavior of fluorescent neutrophils and macrophages were observed after the treatment for 2 h. Photographs of the inflammatory neutrophil migration results were taken with a microscope (MVX10, Olympus, Japan). Dexamethasone (Dex) group was used for positive control.

### Antibiotic susceptibility and checkerboard assays

According to the Clinical and Laboratory Standards Institute (CLSI) guidelines, the minimum inhibitory concentrations (MICs) of the test compounds and colistin against *E. coli* ATCC 25922 (conventional QC strain) and *E. coli* BW25113-mcr-1 (plasmid-mediated colistin-resistant strain) were determined using the broth microdilution method. MIC was defined as the lowest drug concentration that completely inhibited visible bacterial growth. To evaluate compound-colistin interactions, fractional inhibitory concentration (FIC) indices were measured via checkerboard assay. Colistin underwent twofold serial dilution along the x-axis while compounds were diluted along the y-axis, generating a matrix of wells containing combinatorial concentrations. Bacterial suspensions of target strains were inoculated into each well (final density: 5 × 10^5^ CFU mL^−1^). Following 18 h incubation at 37 °C, optical density was measured at 600 nm. The FIC index (FICI) is calculated by dividing the MIC of the drug combination by the MIC of the individual drugs. FIC indices were calculated as: synergism (FICI ≤ 0.5); additive (0.5 < FICI ≤ 1); indifferent (1 < FICI ≤ 2); and antagonism (FICI > 4).

$$FICI = \frac{{MIC_{A}^{combination} }}{{MIC_{A}^{alone} }} + \frac{{MIC_{B}^{combination} }}{{MIC_{B}^{alone} }}$$

### Statistical analysis

Experiments were conducted in triplicate. Data are represented as means ± SEMs or mean ± SD. The statistical significance was evaluated using either a Student's *t*-test or one-way ANOVA for multiple comparisons, conducted via GraphPad Pro software. A *P*-value < 0.05 was set as the threshold for statistical significance.

## Conclusions

In summary, six novel matrine-type alkaloids **(1–6)** featuring previously unreported tricyclic scaffolds were isolated from *S. flavescens* under UV-guided purification. Compounds **1–2** possess a contracted 6/6/5 ring system, while **5** possessed a distinct 6/6/6–5 fused-ring system. A plausible biogenetic pathway was proposed based on a common intermediate. Moreover, several isolates exhibited anti-inflammatory activity, and enhanced the antibacterial potency of colistin against *E. coli*. These findings enrich the chemical diversity of matrine-type alkaloids and provide promising molecular scaffolds for further development of anti-inflammatory and antibacterial adjuvant agents.

## Data Availability

The copies of original UV, IR, CD, HRESIMS, 1D and 2D NMR spectra of compounds **1–6**; Computational ORD/ECD details for compounds **2–6**. This material is available free of charge in the supplementary materials.
